# Supplementary figures and images for: A live cell biosensor protocol for high-resolution screening of therapy-resistant cancer cells
Source: PLoS One. 2026 Feb 17;21(2):e0343016. doi: 10.1371/journal.pone.0343016 (PMC12912615; doi:10.1371/journal.pone.0343016)

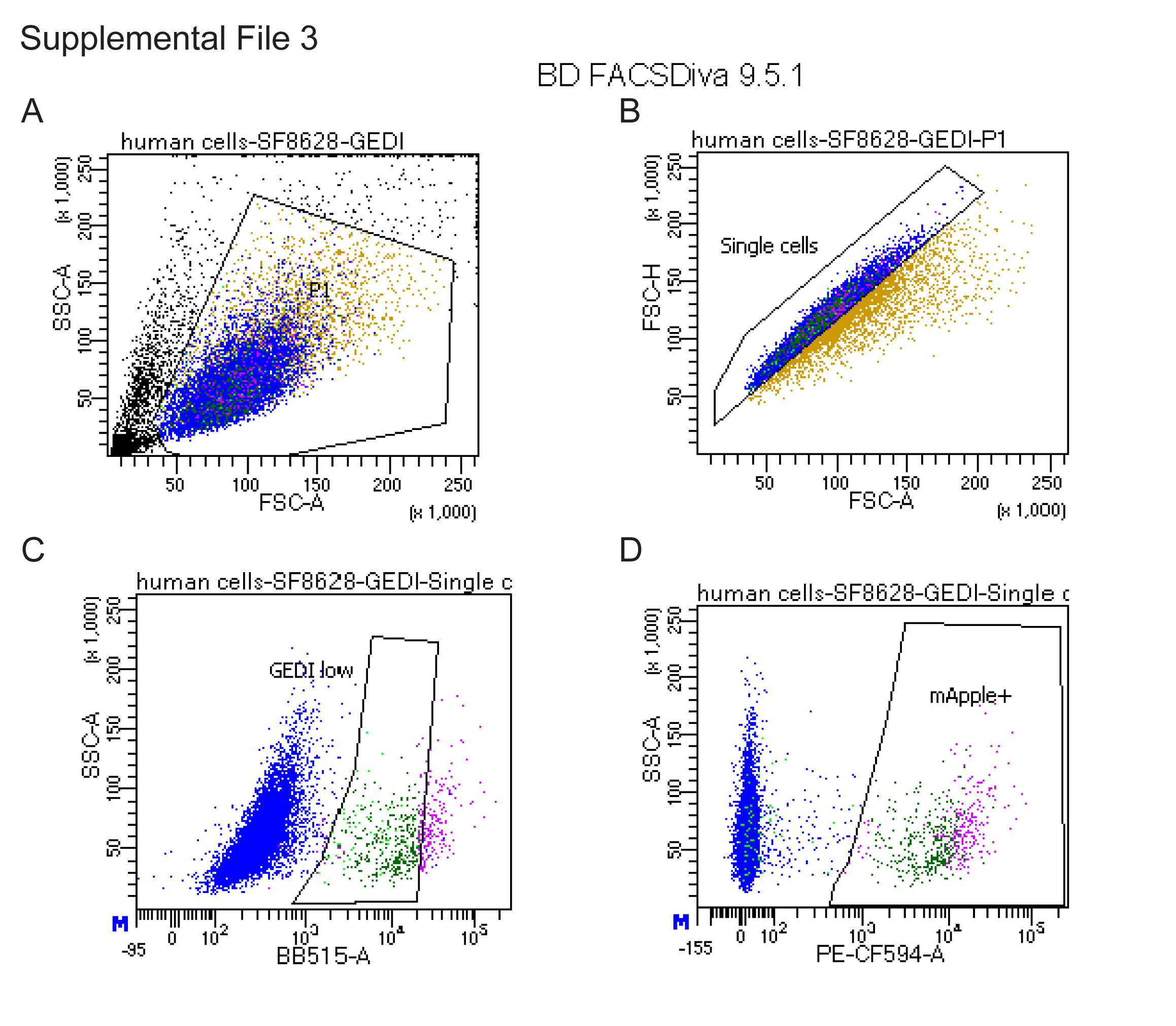

Supplement: S3 Fig — (A) Side scatter area (SSC-A) versus forward scatter area (FSC-A) to exclude debris. (B) Forward scatter height (FSC-H) versus FSC-A gating to select single cells. (C) Gating strategy to exclude GC150-high cells using heat-shocked cells as a positive control, defining a viable GC150-low population for sorting (D) From the GC150-low population, gating strategy to enrich for mApple-positive cells used for downstream experiments. (TIF) [file pone.0343016.s003.tif]

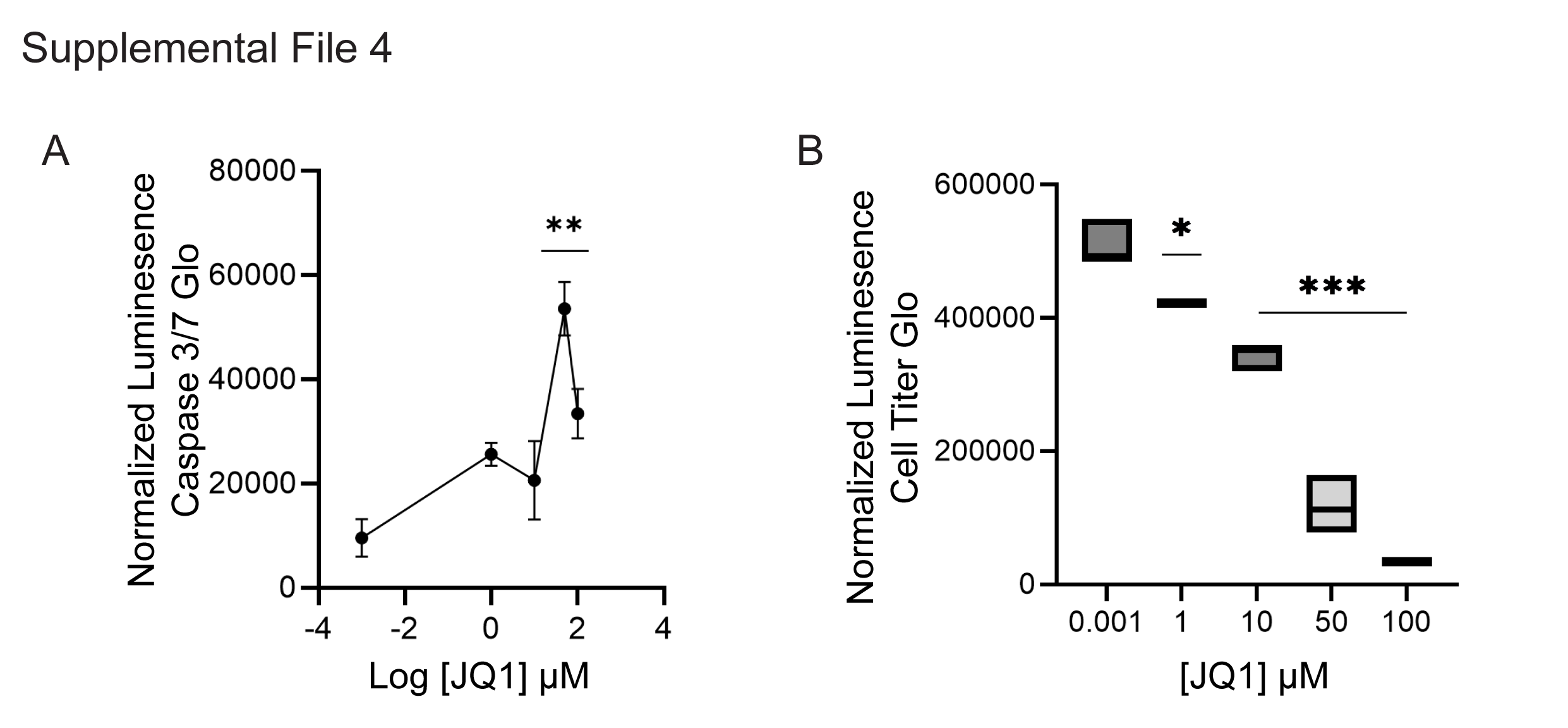

Supplement: S4 Fig — Error bars represent SEM, n = 3. Significance was determined by a one-way ANOVA followed by a Tukey’s post hoc, ** p < 0.01. (B) Normalized CellTiter-Glo luminescence from SF8628 cells treated at increasing JQ1 concentrations and measured 24 hours post-treatment. Error bars represent SEM, n = 3. Significance was determined by a one-way ANOVA followed by a Tukey’s post hoc,* p < 0.05,***p < 0.001. (TIF) [file pone.0343016.s004.tif]
